# Antioxidant activity and cytotoxicity of Jerusalem artichoke tubers and leaves extract on HaCaT and BJ fibroblast cells

**DOI:** 10.1186/s12944-018-0929-8

**Published:** 2018-12-11

**Authors:** Zofia Nizioł-Łukaszewska, Dominika Furman-Toczek, Martyna Zagórska-Dziok

**Affiliations:** University of Information Technology and Management in Rzeszow, Kielnarowa 386a, 36-020 Tyczyn, Poland

**Keywords:** Jerusalem artichoke, Antioxidant activity, ROS, *SOD*-1, *Nox*-4

## Abstract

**Background:**

Extracts from plants, rich in antioxidants may be used as active ingredients of many preparations, mainly due to their antioxidant, regenerative and anti-aging properties. The work involved a comprehensive evaluation of the Jerusalem artichoke (*Helianthus tuberosus L.*) leaf and tuber extract as a multifunctional raw material.

**Methods:**

The plant extracts were prepared by using ultrasound-assisted extraction method (UAE).The content of total phenolic and flavonoid compounds of extracts were determined spectrophotometrically using the Folin-Ciocalteu method and aluminium nitrate nonahydrate, respectively. Antioxidant activity of extract was analyzed using DPPH free radical scavenging assay and the effect of the investigated extracts on the proliferation of keratinocytes (HaCaT) and fibroblasts (BJ) was measured. To detect of intracellular reactive oxygen species level in tested cells, the fluorogenic dye H_2_DCFDA was used. In the next step, the ability of obtained extracts to regulate the expression of genes (*SOD*-1, *Nox*-4) involved in oxidative stress in cells was evaluated.

**Results:**

As a result of the conducted research, it was shown that leaf extract exhibit a higher content of phenols and flavonoids comparing to tuber extracts (5.07 and 7.14 fold higher, respectively). The opposite trend was observed after proliferation assay with Neutral Red test. It was shown that tuber extract in all applied concentrations (25–500 μg·ml^− 1^) had a positive effect on fibroblast growth. The leaf extract showed proliferative activity only for the smallest tested concentrations (25–100 μg·ml^− 1^). Similar trends were observed for HaCaT cells. The distinct effect of leaves and tuber extract on the generation of ROS was observed in HaCaT cells. In the present study, it was shown that tuber and leaf extracts may increase the expression of the ROS *SOD-1* inactivating enzyme gene in the fibroblast cell line. There were no significant differences in gene expression of the ROS *Nox-4* producing enzyme. In the case of keratinocytes, the opposite effect was observed.

**Conclusions:**

The study suggest that Jerusalem artichoke leaves and tubers extracts affect the cell proliferation and can alter the expression of genes related to oxidative stress.

## Background

It is commonly known that oxidative stress caused by free radicals and their derivatives is responsible for disturbing redox homeostasis [1]. It also is one of the primary factors involved in the development of chronic disorders, aging, cancer, degenerative neuronal damage, diabetes mellitus and coronary heart ailment [2, 3]. Reactive oxygen species (ROS) are a group of unstable molecules that are generated in all cells during normal physiological and biochemical processes. Excessive production of free radicals can cause cellular and tissue injury through nonspecific modification and disruption of phospholipids, proteins and nucleic acid [4]. ROS have a negative effect primarily on organelle and cell membranes. Their excess entail peroxidation of membrane lipid and modification of membrane protein. As a result, the structure of the membranes is changing and also their functions are disturbed. Reactive oxygen species causes alternations in cell membrane permeability and fluidity, intracellular enzyme and ion channels activity [5]. These radicals can also may cause DNA damage, leading to mutagenic changes and cell death [6]. Fortunately, well-functioning cells have the ability to defend against the destructive effects of free radicals by the endogenous systems which consist of various enzymes such as superoxidase dismutase (SOD), catalase (CAT) and glutathione peroxide (GPX). An extremely important role in the fight against damage caused by free radicals play antioxidants derived from diet. The rich source of natural antioxidants are primarily plants, mainly fruits, vegetables and herbs, which are now eagerly included in the daily diet. Plant compounds with antioxidant properties can be helpful in the therapy of diseases caused by the action of free radicals [7, 8]. They also can enhance the antioxidant system, reverse oxidative damage and protect against oxidative stress induced deterioration. The antioxidant capacity of plant extracts is mainly associated e.g. with phenolic, flavonoids, isoflavonoids and anthocyanins content. Numerous studies had indicated a correlation between the content of phenolic compounds and flavonoids in plants and the antioxidant activity of plant extracts [9, 10].

*Helianthus tuberosus L*. (Jerusalem artichoke) is a perennial herb from Asteraceae family, originating from the United States where it was cultivated by the indigenous inhabitants. It has a 1,5–3 m tall stem, large leaves, fleshy tubers and yellow sunflower-like flowers [11]. The content of compounds contained in Jerusalem artichoke tubers depends strictly on the harvest conditions and topinambur clones. *Helianthus tuberosus L. tuber*s are a rich source of carbohydrates [12]. It is one of the main sources of inulin in higher plants, its content reaches up to 85% of the dry matter of tubers [13]. The content of free sugars such as glucose, fructose and sucrose is much lower and rarely exceeds 6–8% of dry matter. Proteins present in topinambour tubers reaches up to 10% of dry matter. It contains almost all essentials amino acid such as tryptophan and threonine [11, 14] to the high content of inulin in topinambour tubers, it has been used in folk medicine, and found an application in the treatment of many diseases such as diabetes and rheumatism. This polysaccharide also has a diuretic, aperients, cholagogue, spermatogenic, stomachic and tonic effect [15]. Phytochemical studies demonstrated that topinambour is a source of coumarins, polyacetylenic derivatives, unsaturated fatty acids and sesquiterpenes. Aerial biomass of this plant contain cellulose, hemicelluloses, uronic acids, lignins, proteins and lipids [16, 17]. In addition, due to the presence of many biologically active substances, such as phenolic compounds and flavonoids, leaves of Jerusalem artichoke have also found an application in medicinal purposes [18, 19]. They have been used for treatment of skin wounds, bone fractures, swellings and to reduce pain. These applications are closely related to the activity of *Helianthus tuberosus L.* leaves, which had shown anti-inflammatory, antispasmodic, antimicrobial, antifungal, antipyretic and analgesic effects [19–21].

The aim of our study was to determine the ability of obtained extracts to impact on cell metabolism, influence on the regulation of gene expression (*SOD*-1, *Nox*-4) and to evaluate antioxidants activities of tubers and leaves of *Helianthus tuberosus L.* extracts. The content of flavonoids and phenolic compounds was also determined. These extracts were obtained by ultrasound-assisted extraction method (UAE) and pure ethanol was used as a solvent. It also has been investigated the ability of obtained extracts to impact on cell metabolism and regulate of gene expression (*SOD*-1, *Nox*-4) involved in oxidative stress in cells. The experiments were performed on two human cell lines: keratinocytes (HaCaT) and fibroblasts (BJ).

## Methods

### Plant material and extraction procedure

*Helianthus tuberosus L.* were planted in a sunny position, in the first half of March, every 30 cm in a row. The distance between rows was about 80 cm. The tubers were planted in a depth of 10–15 cm. The leaves and tubers of *Helianthus tuberosus L.* were collected from the region of Subcarpathian Voivodeship in Poland during the September 2017. The collected tubers and leaves were transported to the laboratory and prepared for further analysis. To remove the soil and other impurities, the plant material was cleaned by washing with deionized water. Then, samples of leaves and tubers were used for solvent extraction.

The fresh plant extracts were prepared by using ultrasound-assisted extraction method (UAE). UAE was performed according to the method described by Ying et al. [22] in ultrasonic bath (Digital Ultrasonic Cleaner) equipped with time controller. About 15 g of plant material was packed to the glass tubes and extracted with a 200 ml of ethanol in room temperature. The mixture was homogenized for 50 min (10 cycles for 5 min). Then, obtained extracts were collected and filtered through Whatman filter paper No. 10 and evaporated at 50 °C using a rotary evaporator. Tuber and leaves extracts were stored in the dark in 4 °C for further analysis.

### Total phenolic content determination

The total phenolic content of leaves and tubers *Helianthus tuberosus L.* extracts were determined spectrophotometrically by the Folin-Ciocalteu method according to the procedure reported by Singleton et al. with some modifications [23]. The 300 μL of leaves extract solutions and 1500 μL of 1:10 Folin-Ciocalteau reagent were mixed and after 6 min in the dark, 1200 μL of sodium carbonate (7.5%) was added. After 2 h of incubation in the dark at room temperature, the absorbance at 740 nm was measured spectrophotometrically by AquamateHelion (Thermo Scientific). The total phenolic concentration was calculated from a gallic acid (GA) calibration curve (10–100 mg·mL^− 1^). Data were expressed as gallic acid equivalents (GA)·g^− 1^ of extract averaged from three measurements.

### Total flavonoids content determination

The total flavonoid content of plant extracts were evaluated using aluminium nitrate nonahydrate according to the procedure reported by Woisky and Salatino with modifications [24]. The 600 μL of plant extracts solutions and 2400 μL of mixture (80% C_2_H_5_OH, 10% Al(NO_3_)_3_ × 9H_2_O and 1 M C_2_H_3_KO_2_) were mixed. After 40 min of incubation at room temperature, the absorbance at 415 nm was measured spectrophotometrically by AquamateHelion (Thermo Scientific). The total flavonoids concentration in extracts were calculated from a quercetin hydrate (Qu) calibration curve (10–100 mg·mL^− 1^) and expressed as quercetin equivalents (Qu)·g^− 1^ of extract averaged from three independent measurement.

### DPPH radical scavenging assay

Antioxidant activity of plant extract was analysed using DPPH free radical scavenging assay, according to the method described by Brand-Williams et al. [25]. 167 μL of 4 mM ethanol solution of DPPH was mixed with 33 μL analysed samples in different concentrations (250 μg·ml^− 1^ – 5000 μg·ml^− 1^). The absorbance was measured at λ = 516 nm in every 5 min for 30 min using UV-Vis spectrophotometer Filter Max 5 (Thermo Scientific). DPPH solution mixed with equal volume of distilled water was served as a control. The percentage of the DPPH radical scavenging were calculated using the equation:

$$ \%\mathrm{DPPH}\bullet \mathrm{scavenging}=\left[\mathrm{Abs}\ \mathrm{control}-\mathrm{Abs}\ \mathrm{sample}\right]/\mathrm{Abs}\ \mathrm{control}\times \kern0.37em 100\% $$

### Cell culture

HaCaT cells (normal human keratinocytes, ATCC®) and BJ cells (fibroblasts, ATCC®CRL-2522™) was obtained from the American Type Culture Collection (Manassas, VA 20108, USA). HaCaT cells were maintained in a DMEM (Dulbecco’s modified essential medium, Gibco) with L-glutamine, supplemented with 5% (vol/vol) FBS (fetal bovine serum, Gibco), and 1% (vol/vol) antibiotic (100 U·mL^− 1^Penicillin and 1000 μg·mL^− 1^ Streptomycin, Gibco). Fibroblast were maintained in a MEM (Minimum Essential Medim, Gibco) contains Earle’s salt and L-glutamine, supplemented with 5% (vol/vol) FBS (fetal bovine serum, Gibco), and 1% (vol/vol) antibiotic (100 U·mL^− 1^ Penicillin and 1000 μg·mL^− 1^ Streptomycin, Gibco). All cultured cells were kept at 37 °C in a humidified atmosphere of 95% air and 5% of carbon dioxide (CO_2_).When the cells reached confluence, the culture medium was removed from the flask (VWR) and cells were rinsed two times with sterile PBS (Phosphate-Buffered Saline, Gibco). The confluent layer was trypsinized using Trypsin/EDTA (Gibco) and then resuspended in fresh medium. Cells were treated with varying concentrations (25, 50, 100, 250, 500 μg·mL^− 1^) of ethanolic tuber and leaves topinambour extract suspended in DMSO and its value has not exceed 1%.

### Cell ViabilityAssay

Cell growth was measured using the neutral red dye (Sigma Aldrich). This assay is based on the initial protocol described by Borenfreund et al. and determines the accumulation of the neutral red dye in the lysosomes of viable, uninjured cells [26].

Cells were placed in 96-well plates at a density of 1 × 10^4^ cells per well with fresh medium. After 24 h of pre-culture, medium was aspirated and varying concentrations (25, 50, 100, 250, 500 μg·mL^− 1^) of extracts were added into each well and cultured for another 24 h. The control group were unexposed cells. Following exposure to leaves and tuber extracts, cells were incubated for 2 h with neutral red dye (40 μg·mL^− 1^) dissolved in serum free medium (DMEM or MEM for HaCaT and fibroblasts respectively). After this, cells were washed with Phosphate Buffered Saline (PBS), and then added 150 μL destain solution (EtOH/AcCOOH/H_2_O_2_, 50%/1%/49%) per well, followed by gentle shaking for 10 min, until the neutral red has been extracted from the cells and has formed a homogenous solution. Neutral red dye uptake was determined by measuring the optical density (OD) of the eluted dye at 540 nm in microtiter plate reader spectrophotometer FilterMax F5 (Thermo Fisher). The experiments were performed in triplicates for each extract concentration and presented as percentage of control values.

### Detection of intracellular reactive oxygen species level

To assay the capacity of obtained plant extracts to generate intracellular level of reactive oxygen species in HaCaT and fibroblasts cells, the fluorogenic dye H_2_DCFDA was used. After passively diffusion into the cells, H_2_DCFDA was deacetylated by intracellular esterases into the nonfluorescent compound, that upon oxidation by ROS is converted to the highly fluorescent 2′,7′-dichlorofluorescein (DCF) [27].

Cells were seeded in 96-well plates at a density of 1 × 10^4^cells per well and initially cultured before the experiment for 24 h. After this, culture medium was changed on 10 μM H_2_DCFDA (Sigma Aldrich) in serum-free medium (DMEM or MEM for HaCaT and fibroblasts respectively). Cells were incubated in H_2_DCFDA for 45 min before extracts treatment. Then HaCaT and fibroblast cells were exposed into different extract concentration (25, 50, 100, 250, 500 μg·mL^− 1^), cells treated with 1 mM hydrogen peroxide (H_2_O_2_) was used as a positive control. The DCF fluorescence was measured every 30 min for a total 90 min using a microplate reader FilterMax F5 (Thermo Fisher Scientific) at maximum excitation of 485 nm and emission spectra of 530 nm.

### Real-time PCR for mRNA expression analyses

For the experiment, cell were seeded on 6-well plate at a density 5 × 10^5^ cells per well. After 24 h of pre-incubation, cells were treated with obtained leaves and tuber extracts and incubated for 24 h. Then, samples were collected, and total RNA was extracted from HaCaT and fibroblast cells using a EURx Universal RNA Purification Kit according to the manufacturer’s protocol. The quality and quantity of the mRNA were determined spectrophotometrically at 260 and 280 nm (ND/1000 U*V*/VIS; ThermoFisher NanoDrop). The reverse transcription (RT) reaction was performed at a final volume 20 μL with 1 μg of RNA (as a cDNA template) using the High-Capacity cDNA Reverse Transcription Kit (Applied Biosystem™) according to the manufacturer’s protocol. The RT-PCR run was performed using the Bio Rad C1000 Touch™ Thermal Cycler (Bio Rad Laboratories). The products obtained from the RT reaction were amplified using TaqMan Gene Expression Master Mix (Life Technologies Applied Biosystems) kit with TaqMan probes as primes for the specific genes encoding *Nox-4* (assay ID Hs00276431_m1), *SOD-1* (assay ID Hs00166575_m1), GAPDH (assay ID Hs02786624_g1). Amplification was carried out in a total volume of 20 μL containing 1× TaqMan Gene Expression Master Mix and 1 μL of RT product, which was used as the PCR template. Standard qPCR procedures were performed as follows: 2 min at 50 °C and 10 min at 95 °C, followed by 40 cycles of 15 s at 95 °C and 1 min at 60 °C using an Bio Rad CFX Connect™ Real-Time System (Bio Rad Laboratories).The mRNA expression was calculated relative to a nontargeting control in each experiment. The experiments were repeated in triplicate. The expression level of the gene was calculated using the comparative threshold cycle (Ct) method [28] GADPH was used as the reference gene.

### Statistical analysis

Each value is the mean of three replicates. Values of different parameters were expressed as the mean ± standard deviation (SD). The two-way analysis of variance (ANNOVA) and Bonferroni post-test between groups were performed at the level *P* value of < 0.05 to evaluate the significance of differences between values. Statistical analyses were performed using GraphPad Prism 5.0 (GraphPad Software, Inc., Sand Diego CA).

## Results

In our research the total phenolic content (TPC) and total flavonoid content (TFC) were determined from the calibration curves of gallic acid (y = 0.0046x + 0.0452, R^2^ = 0.9989), and quercetin (y = 0.0153x-0.0053, R^2^ = 0,9996), respectively. The TPC and TFC of tubers and leaves ethanolic extract obtained from ultrasound-assisted extraction are presented in Table 1. The result showed, that leaves extract exhibits the higher phenolic and flavonoid content, compared to the tubers extract (5.07 and 7.14 fold higher, respectively). It also was observed, that the TPC and TFC were increasing in a dose-dependent manner for both extracts.

**Table 1:** Total phenolic and total flavonoid of *Helianthus tuberosus L.* extracts

Total phenolic content (mg GA/g ± S.D.)		Total flavonoids content (mg Qu/g ± S.D.)	
Leaves	Tubers	Leaves	Tubers
389,88 ± 5,24	76,84 ± 4,96	43,20 ± 0,55	6,05 ± 0.32

**Fig. 1:**
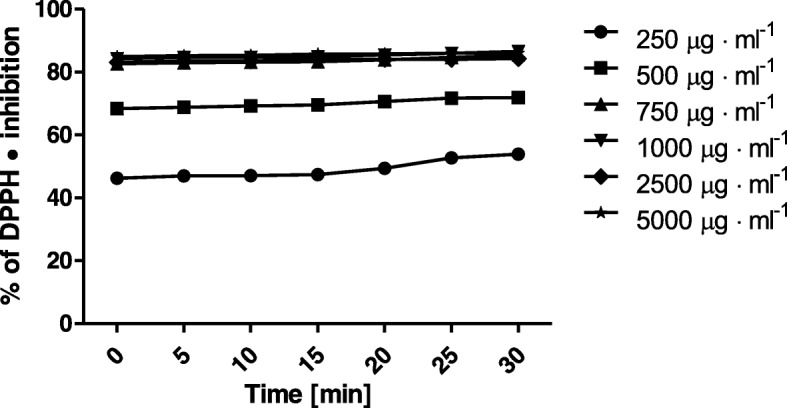
Kinetics of the absorbance changes in DPPH• solutions in the presence of various concentrations of ethanolic extract of *Helianthus tuberosus L.* leaves. Values are mean of three replicate determinations (*n* = 3)

**Fig. 2:**
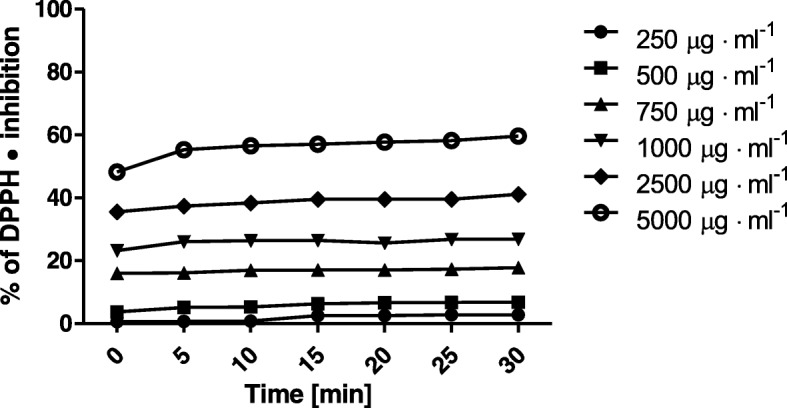
Kinetics of the absorbance changes in DPPH• solutions in the presence of various concentrations of ethanolic extracts of *Helianthus tuberosus L. tuber*s. Values are mean of three replicate determinations (n = 3)

Our research proved, that leaves and tubers of *Helianthus tuberosus L*. are rich source of flavonoids and phenolic compounds. Thus, we hypothesized that both extracts may have an antioxidant potential. The possibility of *Helianthus tuberosus L. tuber* and leaves extract do reduce free radicals was evaluated by DPPH• scavenging assay, where reaction is based on changing colour of free radical solution following to incubation with analysed substances. Decreasing of absorption values after adding tested substances to the radical solution is directly proportional to the number of formed DPPH• [29]. Analyses were conducted in the various concentrations, ranging from 250 μg·mL^− 1^ up to 5000 μg·mL^− 1^. Measurements of extracts antioxidant activity were performed every five minutes over 30 min. Based on obtained data, it was showed that analysed extracts exhibit different antioxidant properties. Jerusalem artichoke leaves extract in all tested concentrations showed higher antioxidant activity, than tuber extract. When considering the lowest used concentration of *H. tuberosus* leaves extract, its ability to reducing DPPH• maintained on 50% level. In the next used concentration (500 μg·mL^− 1^), it was about 10% more, for the others concentrations, percent of scavenged DPPH radical were at the same level. The highest ability (84%) to DPPH• inhibition was observed for the 5000 μg·mL^− 1^ concentration. Considering a leaves extract, we have not observe a dose-dependent response, with the exception of the last lowest tested concentrations (250 and 500 μg·mL^− 1^). For the tubers extract we have noticed a dose-dependent manner in response, up to concentration of 750 μg·mL^− 1^. The lowest concentrations of tested extract have not exhibit antioxidant properties (Fig. 1). Analysed data, obtained from the *H. tuberosus* tuber extract, indicate a much lower ability to scavenge free radicals compared to the Jerusalem artichoke leaves extract. The similar tendency was also observed in case of phenol and flavonoid content. Extracts obtained from leaves was characterized by higher content comparing to the ones obtained from tubers. The highest tested concentration of tuber extract (5000 μg·mL^− 1^) exhibit the ability of radical scavenging over 56% level. The percent of inhibited DPPH• decreased in a dose-dependent manner, for the diluted extract (250 μg•ml^− 1^) it was approximately 0% (Fig. 2). These results are reflected to the total phenolic and total flavonoid concentration in both extracts.

**Fig. 3:**
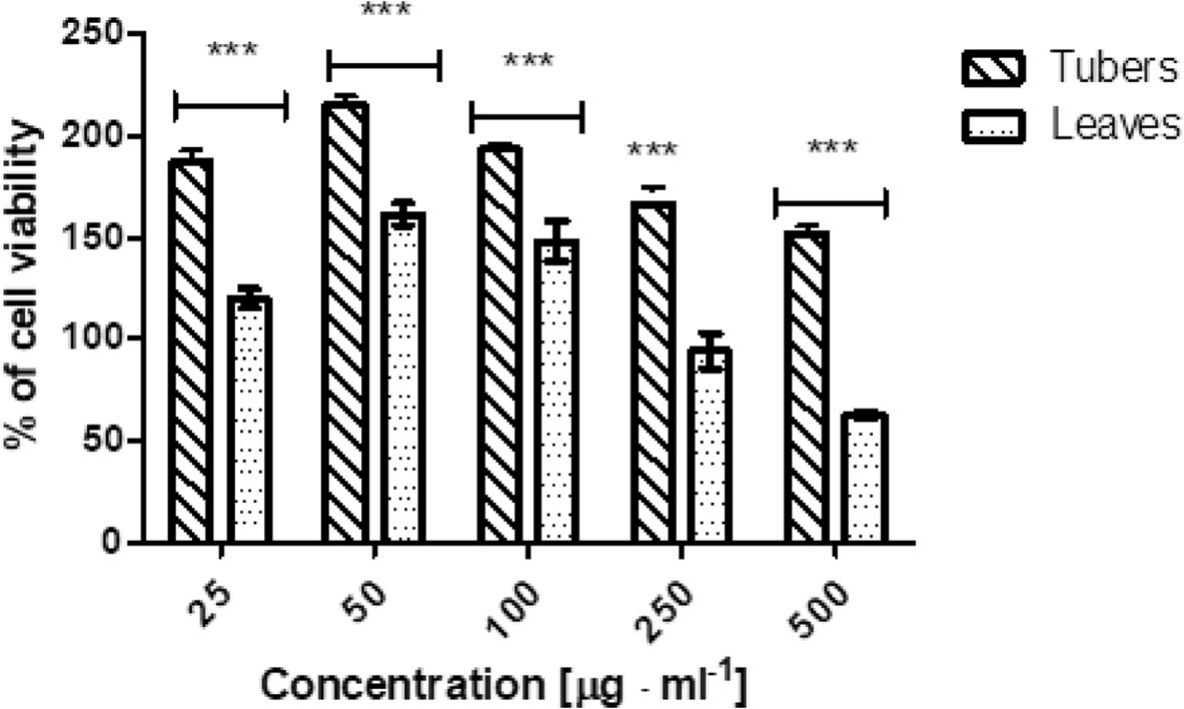
The effect of increasing concentrations of ethanolic *Helianthus tuberosus L. tuber*s and leaves extracts (25, 50, 100, 250, 500 μg·mL^− 1^) on Neutral Red Dye uptake in cultured fibroblast cells after 24 h of exposure. Data are the mean ± SD of three independent experiments, each of which consists of three replicates per treatment group. ***p < 0.001, **p < 0.01, *p < 0.05 versus the control (100%)

**Fig. 4:**
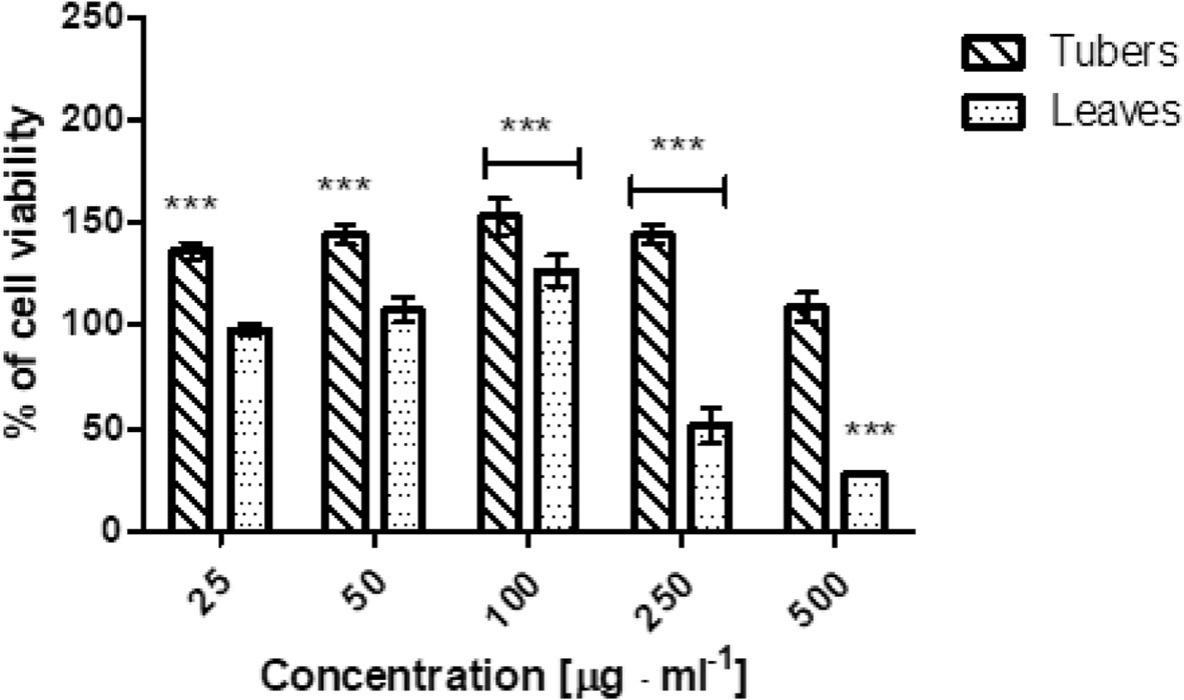
The effect of increasing concentrations of ethanolic *Helianthus tuberosus L. tuber*s and leaves extracts (25, 50, 100, 250, 500 μg·mL^− 1^) on Neutral Red Dye uptake in cultured keratinocytes cells after 24 h of exposure. Data are the mean ± SD of three independent experiments, each of which consists of three replicates per treatment group. ***p < 0.001, **p < 0.01, *p < 0.05 versus the control (100%)

The biological activity of tuber and leaves extracts from *Helianthus tuberosus L.* was also investigated on cells as in vitro model. The cytotoxicity of our extracts was assessed on HaCaT and BJ fibroblast cell lines using neutral red method. Both type of cells were treated with various concentrations of extracts, ranging from 25 to 500 μg·mL^− 1^ for 24 h. The results showed, that the leaves extract displayed a proliferative effects for the smallest tested concentrations, but when doses increased, number of living fibroblast cells decreases up to 62,76% compared with the control group. While, tuber extract in all used concentration showed positive effect on fibroblast growth. The highest proliferative effect on BJ were observed for 50 μg·mL^− 1^ (Fig. 3). Similar results were obtained for HaCaT cells. The smallest concentration of used leaves extract (50 and 100 μg·mL^− 1^) were exhibit proliferative properties, but when dose increased, the cell viability was decreasing. In the highest tested concentration, only 28% of living cells were observed, compared to the control group. Tuber extract in all tested concentration did not show toxic effect on HaCaT cells. Moreover, the induction of proliferative effect of tuber extract was observed. The highest increase of cell proliferation was noticed for 100 μg·mL^− 1^ of tested concentration (Fig. 4). Obtained results clearly suggest that both types of extracts could have both or positive or negative impact on cell proliferative effect. In case of leaves extract these effect might be correlated with high concentration of isolated phenolic and flavonoid compounds. While, in tubers extract considering the low content of these compounds, this behaviour should not be related with them, as happened for leaves. It also was observed, that leaves extract showed less toxicity to BJ cell line, than to HaCaT cells.

**Fig. 5:**
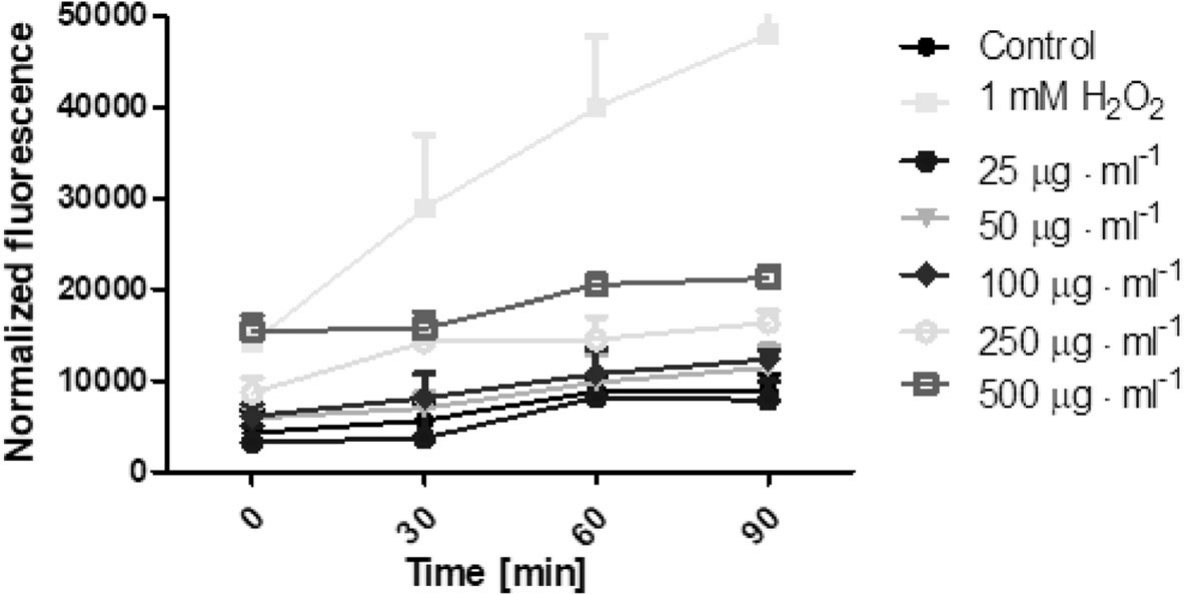
The effect of increasing concentrations ethanolic *Helianthus tuberosus L.* leaves extracts (25, 50, 100, 250, 500 μg·mL^− 1^) on the DCF fluorescence in fibroblast cells. Medium with 1 mM hydrogen peroxide (H_2_O_2_) was used as a positive control. The data are expressed as the mean ± SD of three independent experiments, each of which consisted of three replicates per treatment group

**Fig. 6:**
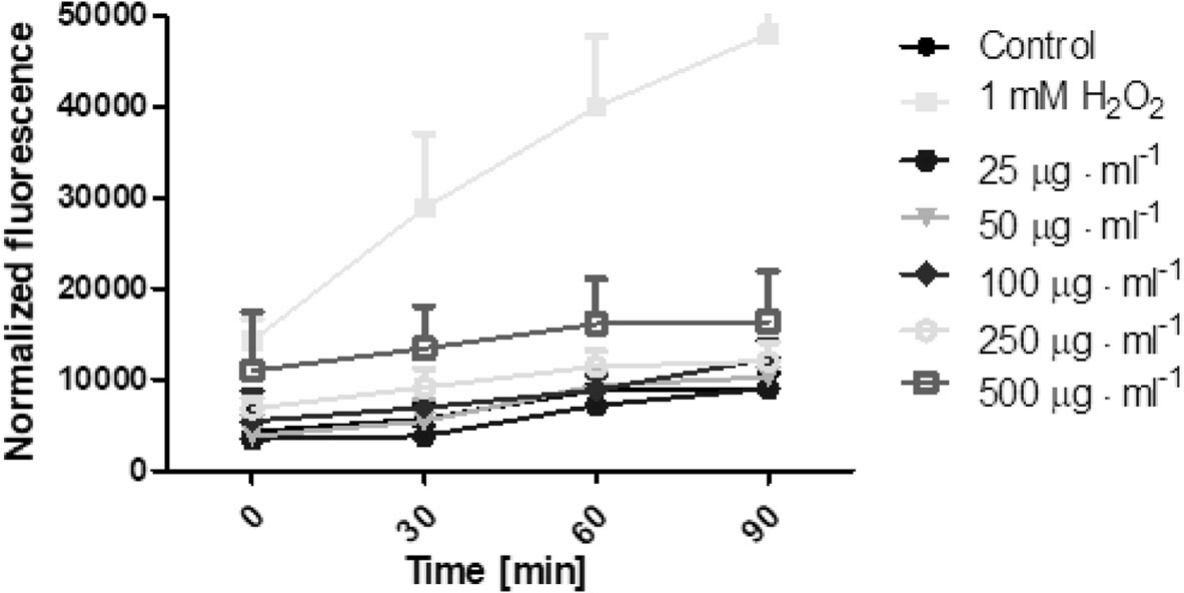
The effect of increasing concentrations ethanolic *Helianthus tuberosus L. tuber*s extracts (25, 50, 100, 250, 500 μg·mL^− 1^) on the DCF fluorescence in fibroblast cells. Medium with 1 mM hydrogen peroxide (H_2_O_2_) was used as a positive control. The data are expressed as the mean ± SD of three independent experiments, each of which consisted of three replicates per treatment group

**Fig. 7:**
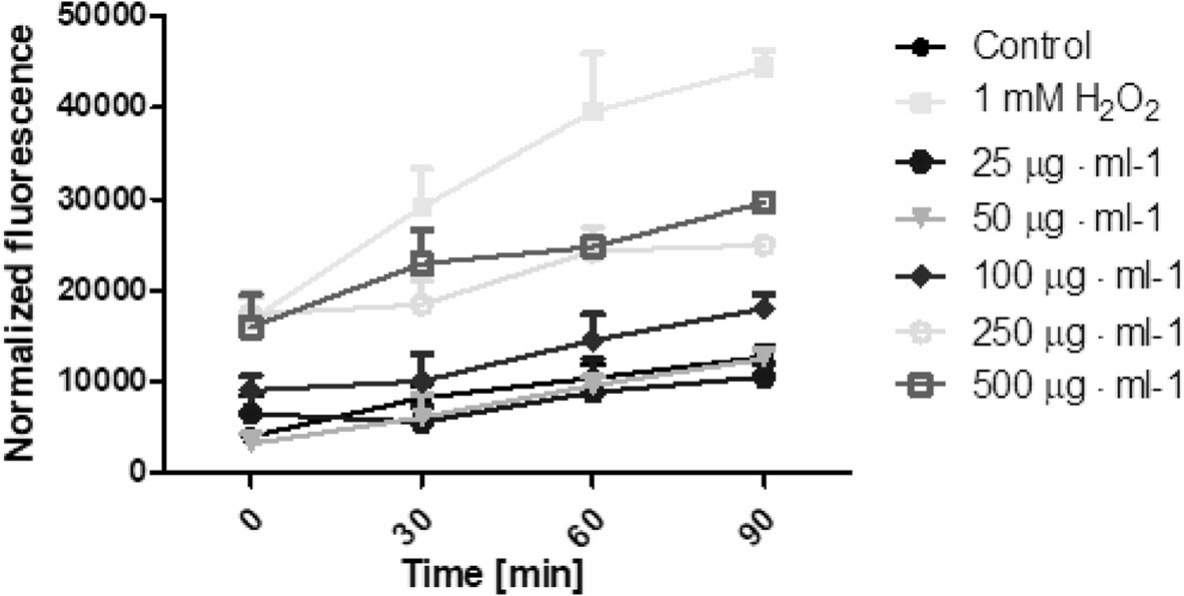
The effect of increasing concentrations ethanolic *Helianthus tuberosus L.* leaves extracts (25, 50, 100, 250, 500 μg·mL^− 1^) on the DCF fluorescence in keratinocytes cells. Medium with 1 mM hydrogen peroxide (H_2_O_2_) was used as a positive control. The data are expressed as the mean ± SD of three independent experiments, each of which consisted of three replicates per treatment group

**Fig. 8:**
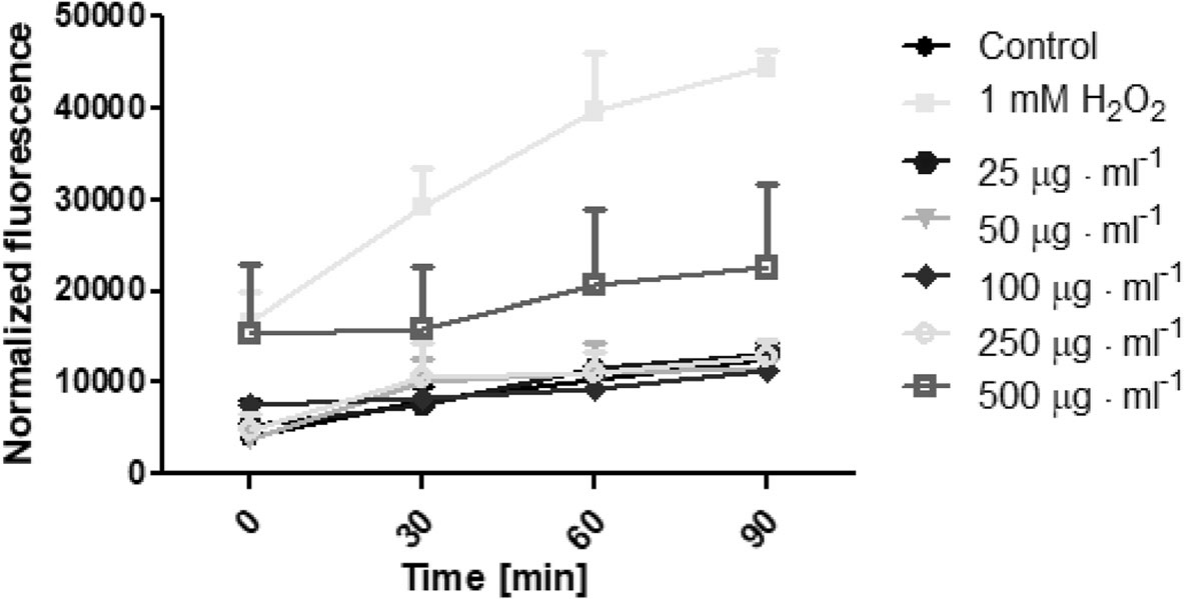
The effect of increasing concentrations ethanolic *Helianthus tuberosus L. tuber*s extracts (25, 50, 100, 250, 500 μg·mL^− 1^) on the DCF fluorescence in keratinocytes cells. Medium with 1 mM hydrogen peroxide (H_2_O_2_) was used as a positive control. The data are expressed as the mean ± SD of three independent experiments, each of which consisted of three replicates per treatment group

The possibility of *Helianthus tuberosus L.* extracts to generate free radicals was evaluated using 2′,7′-dichlorodihydrofluorescein diacetate (H_2_DCFDA) assay. As is recommended [30], we examined whether plant extracts without cells affected the fluorescence of the H_2_DCFDA. Additionally, the separated experiment showed that there were no interactions between plant extracts and H_2_DCFDA substrate in DMEM or MEM medium. During the time of incubation it was shown, that extracts can generate intracellular reactive oxygen species in time- and dose-dependent manner. The biological activity of tested extracts was also specific to the used extract and cell model. Fibroblasts treated with a range of concentration of *Helianthus tuberosus L.* leaves ethanolic extracts showed correlation between used dose and number of ROS generation. When fibroblast cells were treated with low concentrations of these extract, the intracellular ROS level fluctuated at around the control group. With dose above 100 μg·mL^− 1^, reactive oxygen species production were increasing. For the highest used leaves extract concentration, the number of ROS was 2,3 fold higher, compared to the untreated cells (Fig. 5). There was no observed significant differences in tuber extract concentration and ROS production in fibroblast (Fig. 6). A marked effect of leaves and tuber extract on ROS generation was observed in HaCaT cells. The three highest concentrations of leaves extract, significantly increased ROS production. In concentration 500 μg·mL^− 1^, the level of intracellular reactive oxygen species was almost 3 time higher, compared to unexposed cells (Fig. 7). The tuber extract exhibit significant difference in ROS generation merely in the highest tested concentration, where the level of ROS is two times higher, when compared to cells with medium (Fig. 8).

**Fig. 9:**
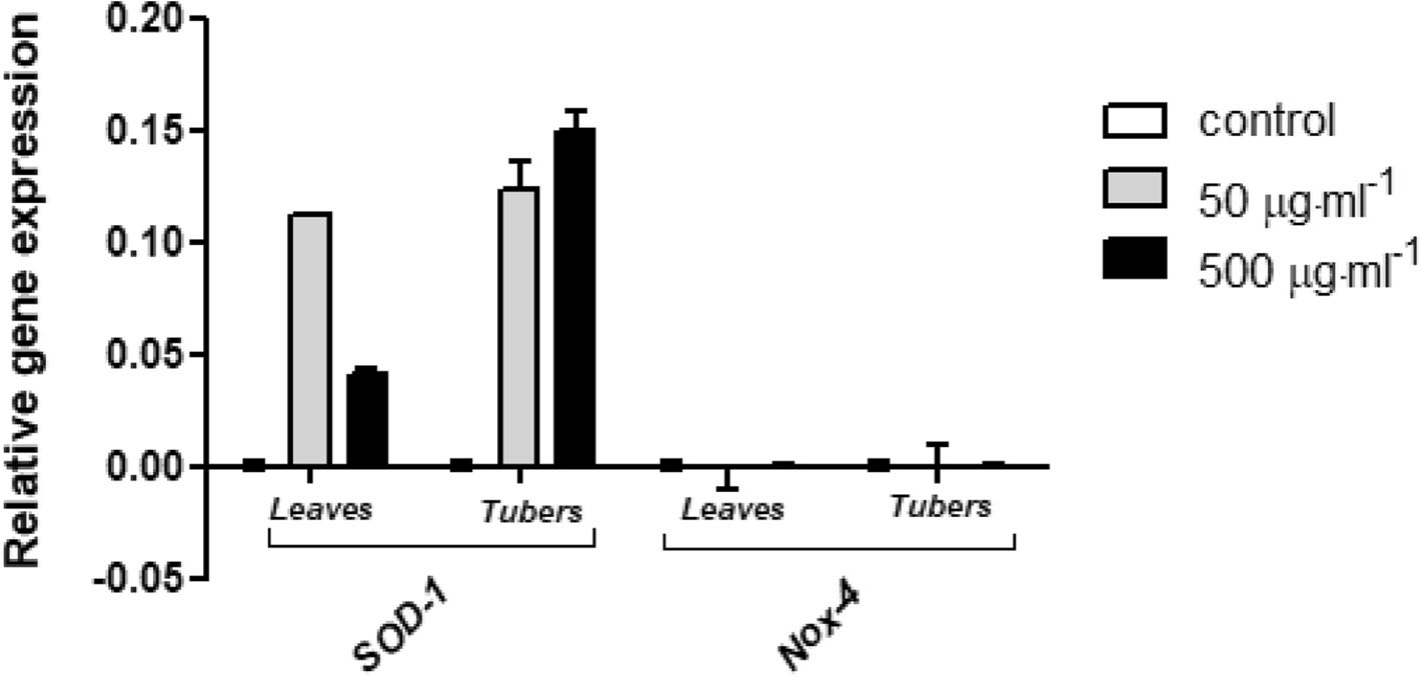
Effects of 50 μg·mL^− 1^and 500 μg·mL^− 1^ ethanolic *Helianthus tuberosus L. tuber*s and leaves extracts on *SOD-1* and *Nox-4* expression in cultured fibroblast cells following 24 h of exposure. mRNA expression was normalized to *GADPH*. The plot is presented in a log2 scale, thus positive values indicate overexpression while negative values indicate underexpression. The data are expressed as the mean ± SD of three independent experiments, each of which consisted of three replicates per treatment group

**Fig. 10:**
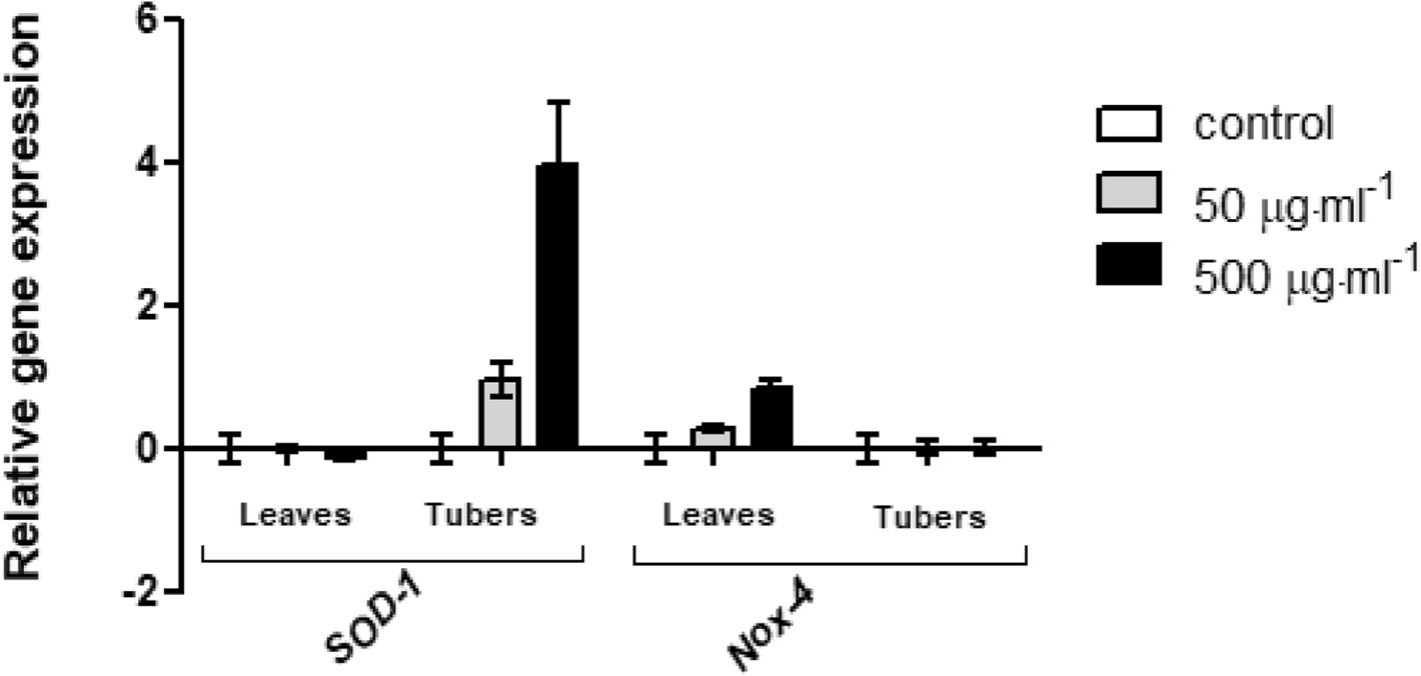
Effects of 50 μg·mL^− 1^ and 500 μg·mL^− 1^ of ethanolic *Helianthus tuberosus L. tuber*s and leaves extracts on *SOD-1* and *Nox-4* expression in cultured keratinocytes cells following 24 h of exposure. mRNA expression was normalized to *GADPH*. The plot is presented in a log2 scale, thus positive values indicate overexpression while negative values indicate underexpression. The data are expressed as the mean ± SD of three independent experiments, each of which consisted of three replicates per treatment group

To examine the impact of tested extracts on ability of expression modulation of two important genes which were known to be involved in oxidative stress the real-time quantitative PCR technique was used. For this experiment two extract concentration were chosen: 50 and 500 μg·mL^− 1^, which showed different effect on cell viability. It have been demonstrated, that both tuber and leaves extracts can increase the gene expression of ROS-inactivating enzyme – SOD-1 in fibroblast cell line. In the case of leaves extract, the higher concentration (500 μg·mL^− 1^) caused a lower *SOD-1* gene expression, which might be correlated with inhibition of cell proliferation and increase of intracellular ROS generation. There was no observed significant differences in the gene expression of ROS-producing enzyme *Nox*-4 (Fig. 9). In turn, in keratinocytes, leaves extract down-regulates *SOD-1* expression and upregulates the expression of *Nox-4*. Addition of the tuber extract to the cell demonstrated opposite effect, it significantly increased *SOD-1* gene expression, whereas *Nox-1* gene was down-regulated (Fig. 10).

## Discussion

Different parts of plants contains various groups of bioactive compounds, including phenols, phenolic acids, flavonoids, isoflavones. All these substances have received attention because of their physiological function, mainly effected by antioxidant, anti-inflammatory and antimicrobial properties [19–21]. The polyphenolic compounds of natural origin, constitute a large group of compounds that are a secondary metabolites of plants. Plants polyphenols are formed by two basic cycles of metabolism, in the shikimic acid cycle and the acetic acid cycle. In the plant material, these substances can be present in the simple form as well as compound with a high degree of complexity. Moreover, many phenolic compounds can occur in a complex with carbohydrates or protein, which have significant impact on their biochemical properties. As the result, the composition of phenolic fraction is highly depended on the species, variety, agrotechnical and climate conditions but most of all on the part of the plant from which the raw material was obtained [31]. Ultrasound-assisted extraction (UAE) seems to be an ideal method, capable to extract high quantities of bioactive compounds in a short time. UAE is also one of economical and efficient technique for isolation of biologically active compounds. Hence, in this work we used UAE method to separate flavonoids and phenolic compounds from leaves and tubers of *Helianthus tuberosus* L. A previous study demonstrates that the leaves and tubers of Jerusalem artichoke contained a high amount of phenolic compound [31, 32]. The tuber are rich source of chlorogenic and dicaffeic acid, in turn as a source of flavonoids leaves contains rutin and camferol. The dominant group of phenolic compounds are isomers of caffeoylquinone and dicavoylquinone. The results obtained in this paper, corresponds with the other research, which indicate on higher phenolic compounds content in leaves than tubers of *Helianthus tuberosus L*. [18, 32]. We were shown that leaf extract exhibit much higher content of phenols and flavonoids comparing to tuber extracts (5.07 and 7.14 fold higher, respectively). One of the most important properties of phenolic compounds derived from plants is their antioxidative activity. The phenols consists of a hydroxyl group and plays an essential role in the antioxidant ability by donating hydrogen and forming stable radical intermediates [33]. The mechanism of action of phenols relies mainly on neutralization of free radicals, chelation of metal ions and induction of dismutase enzymes, as well as peroxidases. Owning to the aforesaid relationship between antioxidant activity and phenolic and flavonoid content, tuber and leaves extracts revealed strong radical scavenging ability may link with their rich phenolic acids concentration. It was also showed, that the ability to DPPH radical scavenging was increasing in a dose-dependent manner for both tested extracts. Moreover, obtained data showed that analysed extracts exhibit different antioxidant properties. It was noticed that leaves extract in all tested concentrations showed higher antioxidant activity, than tuber extract. All analysed concentrations of leaf extract showed more than 40% inhibition of the DPPH radical, whereas only the highest concentration of tuber extract showed similar activity. The much lower ability to scavenge free radicals from the tuber extract, compared to the leaves extract, might depend on differences on phenol and flavonoid content. Yuan et al. (2012) in their paper also showed the high ability of *Helianthus tuberosus L*. leaves extract to scavenging DPPH• and the percent of inhibited radical increased with the concentration increase of the test sample. They also showed, that a higher concentration of 3-O-caffeoylquinic acid and 1,5-dicaffeoyqunic acid (present in the leaves) were the major factors responsible for the radical scavenging activities [18]. Obtained data present a cell-specific and dose-dependent effect of *Helianthus tuberosus L.* leaves extract on cells growth. We have noticed, that the leaves extract exhibit an induction of proliferative effects for the smallest tested concentrations, while increase of concentration caused decrease in number of living fibroblast cells. Considering the tuber extract, it showed positive effect on fibroblast growth in all used concentration. We have observed similar effect of both extracts on HaCaT cells viability. The smallest leaves extract concentration were exhibit proliferative properties, but higher doses decreased the cell viability. Tuber extract in all tested concentrations did not show any toxic effect on HaCaT cells. Moreover, there was observed the induction of proliferation. The positive or negative effect on cell proliferation after treatment of leaves extracts might be correlated with high concentration of isolated phenolic and flavonoid compounds. While in the case of tubers extract, containing the low content of these compounds, this behaviour should be related with other substances isolated during the UAE extraction. For this, the role of tuber extracts from *Helianthus tuberosus L.* on cell proliferation require further and more specific investigation. Zhang et al. [32] in their paper also showed on cell specific toxic activity of Jerusalem artichoke extracts on human lung cancer epithelial cell line (A549) and murine macrophage cell line (RAW 264.7). They exhibited, that A549 ability of cell growth was inhibited, when increased a concentration of tubers extract. In addition, they did not observe any toxic response to RAW 264.7 cell viability at tested concentration. Another research found that extract from leaves of *H. tuberosus* showed cytotoxic activity against breast cancer cell line (MCF-7) and lung cancer cell lines (A549) [31]. Previous conducted studies exhibited on cytotoxic potential of some compounds present in tuber or leaves of Jerusalem artichoke. Research conducted by Pan et al. (2009) indicate on two cytotoxic agents isolated from a methanol extract of the whole plant. Among all isolates only 4,15-isoatriplicolide angelate and 4,15-isoatriplicolide methylacrylate were found to be the most cytotoxic. These compounds are germacranesesquiterpene lactones present only in the leaves of *H. tuberosus* [15].

The possibility of *Helianthus tuberosus L.* extracts to generate free radicals was evaluated using H_2_DCFDA assay. During the time of incubation it was shown, that both types of extracts can generate intracellular reactive oxygen species in time- and dose-dependent manner. The biological activity of tested extracts was also specific to the used extract and cell model. When number of intracellular reactive oxygen species increases, the cells normally triggers its defence system or causes death via apoptotic or necrotic mechanisms. The cellular response to non-physiological level of ROS and oxidative stress might also influence numerous processes including signalling pathways and gene expression regulation [34]. Using the real-time quantitative PCR technique, we examined impact of tested extracts on ability of expression modulation of two important genes which were known to be involved in oxidative stress. The *SOD-1* gene encodes information about enzyme called superoxide dismutase, which the main role is to attach to molecules of copper and zinc to break down superoxide radicals [35]. Another tested gene was *Nox-4*, which encodes a member of the NOX family enzymes, which works as the catalytic subunit the NADPH oxidase complex. The proteins encoded by this gene are responsible for catalyse the reduction of molecular oxygen to different reactive oxygen species [36].

Our results have a direct reflected in data obtained in intracellular reactive oxygen species level detection assay. It has been noticed as well tuber as leaves extracts can induce the *SOD-1* gene expression in BJ cell line. There was no observed any differences between tested groups and control in the *Nox*-4 gene expression. In turn, in case of the HaCaT cells, leaves extracts down-regulated *SOD-1* expression and upregulated the expression of *Nox-4*. On the other hand, tuber extracts demonstrated opposite effect, it upregulated *SOD-1* gene expression, whereas *Nox-1* gene was down-regulated. It suggest that our studies provides evidence, that leaves extract in HaCaT cells is responsible for up-regulation the expression levels of *Nox-4*, that is represent a mechanism for the increase of NADPH oxidase – mediated ROS production by *H. tuberosus L.* leaves extract. While, in fibroblast, no increased intracellular reactive oxygen species was observed after treatment of tuber and leaves extract, which may be the result of up-regulated expression of *SOD-1* gene, which plays an essential role in oxidative stress protection.

## Conclusions

This study demonstrate that the leaves and tubers of Jerusalem artichoke are rich source of phenolic and flavonoid compounds. Leaves contain much higher concentration of those substances compare to the tubers. It might correlate with its strong radical scavenging ability obtained in our research. The radical scavenging was increasing in a dose-dependent manner for both tested extracts. Further investigations showed that both extract, dependent on used doses, can exhibit an induction or inhibition of cell proliferation. We also showed that leaves and tubers extracts can generate intracellular reactive oxygen species in time- and dose-dependent manner. It was also noticed that both types of examined extracts can modulate the SOD-1 and Nox-4 gene expression. The biological activity of tested extracts was also specific to the used extract and cell model.

The high antioxidant capacity of *Helianthus tuberosus* extracts may indicate its potential use as nutriceutics in the treatment of diseases, which are closely related to the excessive production of reactive oxygen species. Due to widespread availability of Jerusalem architoke it may be helpful in preventing various types of diseases.

## Abbreviations

A549: Adenocarcinomic human alveolar basal epithelial cells
BJ: Human fibroblasts
DCF: 2′,7′-dichlorofluorescein
DMEM: Dulbecco’s Modified Eagle Medium
DPPH: 2,2-diphenyl-1-picrylhydrazyl
FBS: Fetal bovine serum
GA: Gallic acid
GAPDH: Glyceraldehyde-3-phosphate dehydrogenase
H_2_DCFDA: 2′,7′-dichlorodihydrofluorescein diacetate
HaCaT: Immortalized human keratinocytes
MCF-7: Human breast adenocarcinoma cell line
MEM: Minimum Essential Media
NADPH: Nicotinamide adenine dinucleotide phosphate
*Nox-4*: Nicotinamide adenine dinucleotide phosphate oxidase 4
OD: Optical density
PBS: Phosphate-buffered saline
Qu: Quercetin hydrate
RAW264.7: Murine macrophage cell line
ROS: Reactive oxygen species
SD: Standard deviation
SOD-1: Superoxide dismutase-1
TFC: Total flavonoid content
TPC: Total phenolic content
UAE: Ultrasound-assisted extraction

## References

[1] Hybertson BM, Gao B, Bose SK, McCord JM (2011). Oxidative stress in health and disease: the therapeutic potential of Nrf2 activation. Mol Asp Med.

[2] Finkel T, Holbrook NJ (2000). Oxidants, oxidative stress and the biology of ageing. Nature.

[3] Sen S, Chakraborty R, Sridhar C, Reddy YRS, De B (2010). Free radicals, antioxidants, diseases and phytomedicines: current status and future prospect. Int J Pharma Sci Rev Res.

[4] Hunt JV, Dean RT, Wolff SP (1988). Hydroxyl radical production and autoxidative glycosylation. Glucose autoxidation as the cause of protein damage in the experimental glycation model of diabetes mellitus and ageing. Biochem J.

[5] Casaril M, Corso F, Corrocher R (1991). Free radicals in human pathology. Recenti Prog Med.

[6] Redza-Dutordoir M, Averill-Bates DA (2016). Activation of apoptosis signalling pathways by reactive oxygen species. Biochim Biophys Acta.

[7] Kancheva VD, Kasaikina OT (2013). Bio-antioxidants - a chemical base of their antioxidant activity and beneficial effect on human health. Curr Med Chem.

[8] Gomes-Rochette NF, Da Silveira Vasconcelos M, Nabavi SM, Mota EF, Nunes-Pinheiro DC, Daglia M, De Melo DF (2016). Fruit as potent natural antioxidants and their biological effects. Curr Pharm Biotechnol.

[9] Orzechowski A, Ostaszewski P, Jank M, Berwid S (2002). Bioactive substances of plant origin in food - impact on genomics. ReprodNutrDev.

[10] Zhao Y, Wu YZ, Wang M, Cheung P, Mehta B (2015). Bioactive substances of plant origin. Handbook of food chemistry.

[11] Ma XY, Zhang LH, Shao HB, Xu G, Zhang F, Ni FT, Brestic M (2011). Jerusalem artichoke (Helianthus tuberosus), a medicinal salt-resistant plant has high adaptability and multiple-use values. J Med Plant Res.

[12] Baldini M, Danusco F, Turi M, Vannozzi GP (2004). Evaluation of new clones of Jerusalem artichoke (Helianthus tuberosus L.) for inulin and sugar yield from stalks and tubers. Ind Crop Prod.

[13] Saengthongpinit W, Sajjaanantakul T (2005). Influence of harvest time and forage temperature on characteristic of inulin from Jerusalem artichoke (Helianthus tuberosus L.) tubers. Postharvest Biol Technol.

[14] Kays SJ, Nottingham SF (2008). Chemical composition and inulin chemistry. Biology and chemistry of Jerusalem artichoke.; CRS press: Boca Raton.

[15] Pan L, Sinden MR, Kennedy AH, Chai H, Watson LE, Graham TL, Kinghorn AD (2009). Bioactive constituents of Helianthus tuberosus (Jerusalem artichoke). Phytochem Lett.

[16] Cabello-Hurtado F, Durst F, Jorin-Novo JV, Werck D (1998). Coumarins in Helianthus tuberosus: characterization, induced accumulation and biosynthesis. Phytochemistry.

[17] Matsuura H, Yoshihara T, Ichihara A (1993). Four new Polyacetylenic glucosides, methyl ,.BETA.-D-GlucopyranosylHelianthenate C-F, from Jerusalem artichoke (Helianthus tuberosus L.). BiosciBiotechnolBiochem.

[18] Yuan X, Gao M, Xiao H, Tan C, Du Y (2012). Free radical scavenging activities and bioactive substances of Jerusalem artichoke (Helianthus tuberosus L.) leaves. Food Chem.

[19] Chen F, Long X, Liu Z, Shao H, Liu L. Analysis of phenolic acids of Jerusalem artichoke (Helianthus tuberosus L.) responding to salt-stress by liquid chromatography/tandem mass spectrometry. ScientificWorldJournal. 2014;568043 10.1155/2014/568043. 10.1155/2014/568043 PMC4181500 25302328

[20] Ahmed MS, El-Sakhawy FS, Soliman SN, Abou-Hussein DMR (2005). Phytochemical and biological study of Helianthus tuberosus L. Egypt J Biomed Sci.

[21] Gengaihi SAE, Enein AMA, Elalla FMA, Baker DHA (2009). Molecular characterization and antimicrobial activities of chicory and Jerusalem artichoke plants. Int J Acad Res.

[22] Ying Z, Han X, Li J (2011). Ultrasound-assisted extraction of polysaccharides from mulberry leaves. Food Chem.

[23] Singleton VL, Orthofer R, Lamuela-Raventos RM (1999). Analysis of total phenols and other oxidation substrates and antioxidants by means of Folin–Ciocalteu reagent. Methods Enzymol.

[24] Matejic JS, Dzamic AM, Mihajilov-Krstev T, Randjelovic V, Krivosej ZD, Marin PD (2012). Total phenolic content, flavonoid concentration, antioxidant and antimicrobial activity of extracts from three Seseli L. taxa. Cent Eur J Biol.

[25] Brand-Williamis W, Cuvelier M, Berset C (1995). Use of a free radical method to evaluate antioxidant activity. LWT Food Sci Technol.

[26] Borenfreund E, Puerner JA (1984). A simple quantitative procedure using monolayer culture for toxicity assays. J Tissue Cult Meth.

[27] Sharma OP, Bhat TK (2009). DPPH antioxidant assay revisited. Food Chem.

[28] Livak KJ, Schmittgen TD (2001). Analysis of relative gene expression data using real-time quantitative PCR and the 2(−Delta DeltaC(T)) method. Methods.

[29] Alam MN, Bristi NJ, Rafiquzzaman M (2013). Review on in vivo and *in vitro* methods evaluation of antioxidant activity. Saudi Pharm J.

[30] Szychowski KA, Wójtowicz AK (2016). TBBPA causes neurotoxic and the apoptotic responses in cultured mouse hippocampal neurons *in vitro*. Pharmacol Rep.

[31] Yuan X, Gao MZ, Wang K, Xiao HB, Tan CY, Du YG (2008). Analysis of chlorogenic acids in Helianthus tuberosus Linn leaves using high performance liquid chromatography–mass spectrometry. Se Pu.

[32] Zhang Q, Kim HY (2015). Antioxidant, anti-inflammatory and cytotoxicity on human lung epithelial A549 cells of Jerusalem artichoke (Helianthus tuberosus L.) tuber. Korean J Plant Resour.

[33] Fukumoto LR, Mazza G (2000). Assessing antioxidant and prooxidant activities of phenolic compounds. J Agric Food Chem.

[34] Martindale JL, Holbrook NJ (2002). Cellular response to oxidative stress: signaling for suicide and survival. J Cell Physiol.

[35] Zelko IN, Mariani TJ, Folz RJ (2002). Superoxide dismutase multigene family: a comparison of the CuZn-SOD (SOD1), Mn-SOD (SOD2), and EC-SOD (SOD3) gene structures, evolution, and expression. Free RadicBiol Med.

[36] Guo S, Chen X (2015). The human Nox4: gene, structure. physiological function and pathological significance J Drug Target.

